# Extraction of Gold Based on Ionic Liquid Immobilized in UiO-66: An Efficient and Reusable Way to Avoid IL Loss Caused by Ion Exchange in Solvent Extraction

**DOI:** 10.3390/molecules28052165

**Published:** 2023-02-25

**Authors:** Xinyu Cui, Yani Wang, Yanfeng Wang, Pingping Zhang, Wenjuan Lu

**Affiliations:** Institute of Materia Medica, Shandong First Medical University & Shandong Academy of Medical Sciences, Jinan 250062, China

**Keywords:** ionic liquids, UiO-66, gold recovery, regenerability, selective adsorption

## Abstract

Ionic liquids (ILs) have received considerable attention as a promising green solvent for extracting metal ions from aqueous solutions. However, the recycling of ILs remains difficult and challenging because of the leaching of ILs, which is caused by the ion exchange extraction mechanism and hydrolysis of ILs in acidic aqueous conditions. In this study, a series of imidazolium-based ILs were confined in a metal–organic framework (MOF) material (UiO-66) to overcome the limitations when used in solvent extraction. The effect of the various anions and cations of the ILs on the adsorption ability of AuCl_4_^−^ was studied, and 1-hexyl-3-methylimidazole tetrafluoroborate ([HMIm]^+^[BF_4_]^−^@UiO-66) was used for the construction of a stable composite. The adsorption properties and mechanism of [HMIm]^+^[BF_4_]^−^@UiO-66 for Au(III) adsorption were also studied. The concentrations of tetrafluoroborate ([BF_4_]^−^) in the aqueous phase after Au(III) adsorption by [HMIm]^+^[BF_4_]^−^@UiO-66 and liquid–liquid extraction by [HMIm]^+^[BF_4_]^−^ IL were 0.122 mg/L and 18040 mg/L, respectively. The results reveal that Au(III) coordinated with the N-containing functional groups, while [BF_4_]^−^ was effectively confined in UiO-66, instead of undergoing anion exchange in liquid–liquid extraction. Electrostatic interactions and the reduction of Au(III) to Au(0) were also important factors determining the adsorption ability of Au(III). [HMIm]^+^[BF_4_]^−^@UiO-66 could be easily regenerated and reused for three cycles without any significant drop in the adsorption capacity.

## 1. Introduction

Ionic liquids (ILs) represent a class of environmentally friendly “green” solvents with unusual physical and chemical properties, such as a low vapor pressure and the absence of volatilization, and they can be used for the extraction of metal ions from aqueous solutions [1,2,3,4,5,6,7,8,9]. The most commonly proposed mechanism for the extraction of metal ions into the hydrophobic ionic liquid phase is ion exchange [2,3,4,5,6,7,8,9]. The simultaneous release of IL ions to the aqueous phase (resulting in IL losses and aqueous phase pollution) when metal ions are extracted into the IL phase has been reported in many cases. Moreover, the chemical stability of ILs has already aroused attention. The hydrolysis of fluorine-based anions of ILs has been reported. Fernandes and co-workers found that the hydrolysis of [BF_4_]^−^ even occurred at room temperature [10]. The hydrolysis of fluorine-based anions generates an abundance of toxic hydrofluoric acid (HF), which results in environmental pollution.

Supported ionic liquids (SILs) are a new type of solid material prepared through physical adsorption, chemical bonding, or loading of ILs onto porous supports, and they have the characteristics of both ILs and carriers. The process can significantly improve the utilization of ILs, solve the problems of the high viscosity, separation, and mass transfer of ILs, and expand the application field of ILs. Various porous materials have been used as supports, such as covalent organic frameworks [11], porous celluloses [12,13], molecular sieves [14], magnetic materials [15], resins [16], porous silica [17], and biopolymers [18]. SILs have been widely used in various applications, such as catalysis [19], adsorption separation [12,13,15,16,17,18], gas storage [14,20], and electrode materials [11]. However, the ion exchange mechanism is responsible for the adsorption of metal ions in SILs in many cases [13,16,17,18].

Immobilizing ILs in metal–organic frameworks (symbolized as IL@MOFs) is favorable for the adsorption of metal ions from the aqueous phase because of the strong bond between ILs and MOFs, as reported in [19,21,22]. UiO-66 is a porous crystalline material with Zr^2+^ as the excess metal ion and terephthalic acid as the organic ligand. The Zr_6_ cluster ([Zr_6_O_4_(OH)_4_]) has 12 terephthalic acids (H_2_BDC), the maximum number of organic ligands and metal coordination clusters in MOFs. Compared with other MOFs, the dense structural unit and Zr-O property make UiO-66 the most stable porous material in aqueous solutions and other common solvents [23,24,25,26,27]. It possesses high thermal and mechanical stability, as well as good resistance to strong acids [28,29]. Moreover, UiO-66 contains tetrahedral and octahedral pore cages, and this 3D pore structure facilitates the dispersion of ILs [30]. The [Zr_6_O_4_(OH)_4_] metal ion clusters of UiO-66 are easily approached by the spherical anions of ILs, such as [BF_4_]^−^ and hexafluorophosphate ([PF_6_]^−^) [31]. Therefore, UiO-66, with a stable and well-defined structure, becomes a promising host material for immobilizing IL anions.

Gold is valuable, and the recovery of Au(III) from waste CPUs has become attractive due to the limited availability of gold. Gold can be extracted from electronic waste technology via pyrometallurgy and hydrometallurgy [32]. The selectivity of pyrometallurgy is poor, the process is complex, the cost is high, and the generation of volatile metals and dust causes environmental pollution. Therefore, hydrometallurgy is the main method used to leach metals from e-waste [33]. Currently, commonly used methods for gold recovery from e-waste leaching solutions include solvent extraction [4,34], chemical precipitation [35], membrane separation [36], and adsorption [37,38,39]. In contrast, adsorption has the advantages of mild conditions, simple operation, low cost, and recyclability. Adsorption is the most economical and practical method for gold recovery, with great potential. Additionally, in solvent extraction, it is worth noting that ILs themselves extract [AuCl_4_]^−^ through anion exchange. Based on the above, a study of gold recovery from waste CPUs using composite materials composed of ILs encapsulated in UiO-66 (ILs@UiO-66) was conducted. The composites combine the excellent physicochemical properties of ILs and the advantages of the high surface area and high porosity of UiO-66. However, the investigations on ILs@UiO-66 composites available thus far are mainly focused on examining the effect of the loading of ILs on their extraction performance, while the effects of stronger interactions of IL anions with UiO-66 are rarely explored. Therefore, in the current study, an effort was made to address this issue. We expect that the knowledge gained will not only contribute to a better understanding of the properties of ILs@UiO-66 composites for gold recovery, but will also be beneficial in providing guidance on how to overcome the limitations of ILs used in acidic conditions.

## 2. Results

### 2.1. IL Dependence of Adsorption Ratio

In this study, UiO-66, originally synthesized by Lillerud and co-workers [40], was used as a support for ILs. Previous studies have shown that UiO-66 is one of the most stable MOFs. It possesses high thermal and mechanical stability, as well as good resistance to strong acids [28]. The unique robustness of UiO-66 derives from the 12-coordinated Zr_6_ clusters that constitute the framework. It is built up from [Zr_6_O_4_(OH)_4_] units interlinked via terephthalate linkers, leading to a 3D porous framework with two types of cages, namely, tetrahedral and octahedral cages, with diameters of 8 and 11 Å, respectively [22].

**Figure 1:**
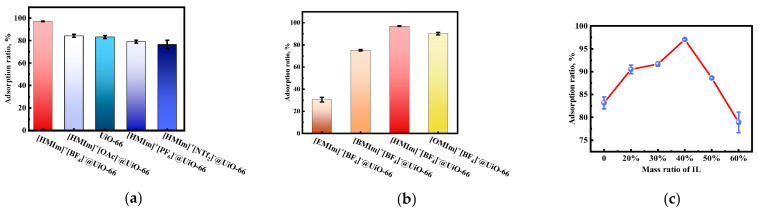
(**a**) IL cation dependence of adsorption ratio; (**b**) IL anion dependence of adsorption ratio (**c** _Au(III)_ = 60 mg/L, V _Au(III)_ = 10 mL, m _[HMIm]+[BF4]-@UiO-66_ = 10 mg, t = 6 h, T = 35 °C, pH = 2); (**c**) mass ratios of [HMIm]^+^[BF_4_]*^−^* (c _Au(III)_ = 60 mg/L, V _Au(III)_ = 10 mL, m _[HMIm]+[BF4]-@UiO-66_ = 10 mg, t = 6 h, T = 35 °C, pH = 2).

In order to select ionic liquids with an appropriate structure, the influence of the anions and cations of ILs on the Au(III) adsorption ability of ILs@UiO-66 was examined at first. Several ILs were transported into UiO-66, and their Au(III) adsorption ability was investigated, including [HMIm]^+^-based ILs with different anions (tetrafluoroborate [BF_4_]*^−^*, hexafluorophosphate [PF_6_]*^−^*, acetate [OAc]*^−^*, and bistrifluoromethane sulfonimide [NTf_2_]*^−^*) and [BF_4_]*^−^*-based ILs with different cations (1-ethyl-3-methylimidazolium [EMIm]^+^, 1-butyl-3-methylimidazole [BMIm]^+^, [HMIm]^+^, and 1-octyl-3-methylimidazolium [OMIm]^+^). The results are shown in Figure 1a, b.

According to the reported studies, the composition of ILs is an important factor that affects the extractability of AuCl_4_*^−^* when ILs are used in solvent extraction or immobilized on solid supports. The extractability of AuCl_4_*^−^* was found to be greater for ILs composed of more hydrophilic anions and more hydrophobic cations [13,41]. In this work, with the [HMIm]^+^ cation, the Au(III) adsorption ability of ILs/UiO-66 was in the order of [BF_4_]*^−^* > [OAc]*^−^* > [PF_6_]*^−^* > [NTf_2_]*^−^*, while with the [BF_4_]*^−^* anion, it was in the order of [HMIm]^+^ > [OMIm]^+^ > [BMIm]^+^ > [EMIm]^+^. These results are roughly related to the hydrophilicity of the anions and hydrophobicity of the cations. The hydrophilicity of the used anions in our work decreased in the order of [OAc]*^−^* > [BF_4_]*^−^* > [PF_6_]*^−^* > [NTf_2_]*^−^*, and the hydrophobicity of the cations decreased in the order of [OMIm]^+^ > [HMIm]^+^ > [BMIm]^+^ > [EMIm]^+^. Therefore, ILs composed of more hydrophilic anions and hydrophobic cations are more conducive to adsorbing Au(III), except for [OAc]*^−^*, [BF_4_]*^−^*, [OMIm]^+^, and [HMIm]^+^.

For [OAc]*^−^* and [BF_4_]*^−^*, the quasi-spherical structure of the [BF_4_]*^−^* anion allows it to fit more closely in the Zr sites of UiO-66 than [OAc]*^−^*, which has a chain-like structure [31]. As a result of this, the Au(III) adsorption ability showed the order of [HMIm]^+^[BF_4_]*^−^*@UiO-66 > [HMIm]^+^[OAc]*^−^*@UiO-66. Hence, the effect of anions on the adsorption ratio of Au(III) resulted from the hydrophilic effect in combination with the anionic structure.

For [OMIm]^+^ and [HMIm]^+^, considering the very large [OMIm]^+^ cation transported into UiO-66 cavities, it is possible to block the pores of UiO-66, resulting in difficult Au(III) adsorption [42]. Thus, the effect of cations on the adsorption ratio of Au(III) was caused by the hydrophobic effect in combination with the cationic size. Since [HMIm]^+^[BF_4_]*^−^*@UiO-66 showed the best adsorption result, the rest of the study was carried out with this adsorbent.

Subsequently, the influence of [HMIm]^+^[BF_4_]*^−^* IL loading on the adsorption ratio of Au(III) was investigated (by conducting experiments on the synthesis of [HMIm]^+^[BF_4_]*^−^*@UiO-66 with different mass ratios of [HMIm]^+^[BF_4_]*^−^* to UiO-66). UiO-66 was loaded with [HMIm]^+^[BF_4_]*^−^* from [HMIm]^+^[BF_4_]*^−^*-ethanol solutions. As reported, the IL mass in the solution is essentially proportional to the IL loading in the MOF pores [43]. Thus, the investigations of the effect of [HMIm]^+^[BF_4_]*^−^* loading were conducted by varying the mass ratio of [HMIm]^+^[BF_4_]*^−^* to UiO-66 in ethanol solutions. As shown in Figure 1c, with an increase in the mass ratio of [HMIm]^+^[BF_4_]*^−^*, the adsorption ratio of Au(III) increased at first and then decreased when the mass ratio was higher than 40%. The maximum adsorption ratio maintained above 95% was achieved at medium loadings, when the mass ratio of [HMIm]^+^[BF_4_]*^−^* was 40%. At low [HMIm]^+^[BF_4_]*^−^* loadings, incorporating more ILs into UiO-66 provided more sites that can interact with Au(III); thus, the adsorption ability increased. At high [HMIm]^+^[BF_4_]*^−^* loadings, where the percentage of the free volume of the adsorbent decreased when more ILs were transported into UiO-66, the pore blockage hindered the passage of gold ions [16,43].

### 2.2. Characterization of [HMIm]^+^[BF_4_]^−^@UiO-66

The resulting immobilized [HMIm]^+^[BF_4_]*^−^* in UiO-66 was characterized by various microscopic and spectroscopic techniques, such as scanning electron microscopy (SEM), Fourier transform infrared spectra (FT-IR), and X-ray diffraction spectrometry (XRD). The specific surface area and porous structures of UiO-66 and [HMIm]^+^[BF_4_]*^−^*@UiO-66 were investigated via nitrogen adsorption–desorption isotherms and pore size distribution curves.

The surface morphology of UiO-66 before and after IL loading was observed via SEM. Figure S1a, b show the SEM images of UiO-66 and [HMIm]^+^[BF_4_]*^−^*@UiO-66, indicating that no detectable IL phase formed on the surface of UiO-66 [42]. A large number of irregularly rounded particles can be found closely attached to the characterized surfaces of UiO-66 and [HMIm]^+^[BF_4_]*^−^*@UiO-66, and this rough surface can enhance the adsorption capacity. This is the same form as previously reported for UiO-66 [25]. At the same time, many pores and voids can be observed in Figure S1, and this porous tertiary structure is beneficial for the following adsorption performance. The dimensions of both samples are the same, indicating that the morphological characteristics of the samples are the same before and after synthesis.

**Figure 2:**
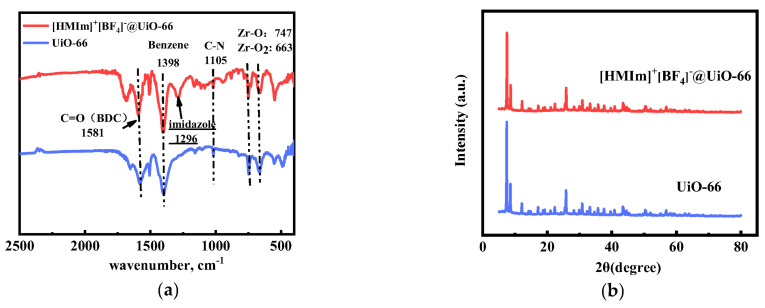
(**a**) FT-IR spectra of UiO-66 and [HMIm]^+^[BF_4_]*^−^*@UiO-66; (**b**) PXRD patterns of UiO-66 and [HMIm]^+^[BF_4_]*^−^*@UiO-66.

To confirm the successful immobilization of [HMIm]^+^[BF_4_]*^−^* in UiO-66, FT-IR was performed. The FT-IR spectra of UiO-66 and [HMIm]^+^[BF_4_]*^−^*@UiO-66 are shown in Figure 2a. The band at 1581 cm*^−^*^1^ in the spectra of UiO-66 and [HMIm]^+^[BF_4_]*^−^*@UiO-66 is due to the stretching vibration of the C=O bond on the BDC, the appearance of the characteristic band at 1398 cm*^−^*^1^ indicates the typical framework vibration of the benzene ring, and the stretching vibrations of the Zr-O and Zr-O_2_ bonds are indicated by the bands at 747 cm*^−^*^1^ and 663 cm*^−^*^1^ [26,27], which indicate the successful synthesis of UiO-66. The strong band at 1105 cm*^−^*^1^ can be attributed to C-N stretching, and the band at 1296 cm*^−^*^1^ can be attributed to the imidazole ring stretching [42], which indicates the successful sequestration of IL onto UiO-66.

The powder XRD patterns of UiO-66 and [HMIm]^+^[BF_4_]*^−^*@UiO-66 are shown in Figure 2b. The prominent characteristic peaks of UiO-66 are located at 2θ = 7.5°, 8.5°, and 25.8°, which are consistent with the UiO-66 patterns in the literature [20,26,29,42]. In addition, [HMIm]^+^[BF_4_]*^−^*@UiO-66 has the same typical peaks and intensities, indicating that the IL-supported UiO-66 crystal pore cage structure remains intact. However, there are no characteristic peaks of [HMIm]^+^[BF_4_]*^−^* in the XRD pattern of [HMIm]^+^[BF_4_]*^−^*@UiO-66, probably because [HMIm]^+^[BF_4_]*^−^* is entrapped in the framework of UiO-66 and there is no crystalline phase of [HMIm]^+^[BF_4_]*^−^* to be detected.

**Figure 3:**
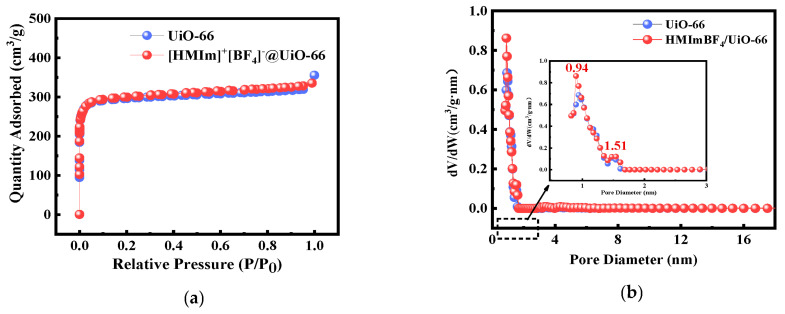
(**a**) Nitrogen adsorption–desorption isotherms of UiO-66 before and after [HMIm]^+^[BF_4_]*^−^* modification; (**b**) aperture distribution curve of UiO-66 before and after [HMIm]^+^[BF_4_]*^−^* modification.

The N*_2_* adsorption–desorption isotherms of the adsorbent materials are shown in Figure 3a. The characteristics of UiO-66 and [HMIm]^+^[BF_4_]*^−^*@UiO-66 both follow an I-shaped curve [27], characteristic of microporous materials, proving the existence of microporosity in the materials. IL curing can still maintain the microporous properties of UiO-66, and the stable microporous structure benefits the diffusion of Au(III). The pore size distribution parameters were obtained based on the Brunauer–Emmett–Teller (BET) pore size distribution curves. The BET surface area and pore volume of UiO-66 and [HMIm]^+^[BF_4_]*^−^*@UiO-66 were 1211.178 m^2^/g and 0.455 cm^3^/g, and 1207.26 m^2^/g and 0.442 cm^3^/g, respectively. Before and after IL curing, the high specific surface area possessed by UiO-66 can provide more adsorption sites for Au(III). In addition, the pore diameters of [HMIm]^+^[BF_4_]*^−^*@UiO-66 are mainly distributed around 0.94 nm and 1.51 nm. These diameters are much larger than those of gold species. Thus, this facilitates the diffusion of gold ions from the surface of the material into the pores, so that there are enough adsorption sites on the surface to adsorb gold ions. The reduction in the specific surface area and pore volume of UiO-66 before and after loading also confirms that the IL was successfully transported into UiO-66.

### 2.3. Adsorption Mechanism

#### 2.3.1. The Anion Influence on Adsorption

In liquid–liquid extraction, ILs extract [AuCl_4_]^−^ through the anion exchange mechanism [3,4,5,6,7,8,44]. A series of SILs as adsorbents have been developed; however, the anion exchange mechanism is responsible for the adsorption of [AuCl_4_]^−^ [13,16,18]. The loss of IL anions not only leads to water pollution, but also challenges the regeneration of ILs.

By introducing an IL into the porous framework of an MOF, it has been reported that the interionic interactions become stronger due to the interaction of the anions of the IL with the metal sites of the MOF, and the direct interaction between the imidazolium ring of the IL with either the MOF or the anion of the IL [21]. Another computational study concluded that anions of ILs ([NTf_2_]^−^, [PF_6_]^−^, [BF_4_]^−^, and [SCN]^−^) have a stronger interaction than [BMIm]^+^ cations with MOFs. Thus, UiO-66 possesses application potential in avoiding the anion exchange mechanism because of the interactions between IL anions and the Zr atoms of UiO-66.

ILs with [PF_6_]^−^ or [NTf_2_]^−^ anions are more widely used in the liquid–liquid extraction of metal ions, despite their higher cost compared to ILs with [BF_4_]^−^ anions [1,2,4,5,6,7,8]. The applications of [BF_4_]^−^ ILs in the extraction of metal ions are limited due to the higher water miscibility compared to ILs with [PF_6_]^−^ or [NTf_2_]^−^. To figure out whether UiO-66 can effectively confine [BF_4_]^−^ to restrain the exchange process of [BF_4_]^−^ with [AuCl_4_]^−^ and the dissolution of [HMIm]^+^[BF_4_]^−^ in water, the concentrations of [BF_4_]^−^ in the aqueous phase after [AuCl_4_]^−^ adsorption or liquid–liquid extraction were quantified. An amount of 150ppm of [AuCl_4_]^−^ at pH = 2 was used for the study at first. The adsorption of [AuCl_4_]^−^ by [HMIm]^+^[BF_4_]^−^@UiO-66 and the liquid–liquid extraction of [AuCl_4_]^−^ by [HMIm]^+^[BF_4_]^−^ IL itself were performed under the same conditions (c _Au(III)_ = 60 mg/L, V _Au(III)_ = 10 mL, T = 35 °C, t = 6 h). The ion chromatograms for [BF_4_]^−^ in the aqueous phase are shown in Figure S3. When the adsorption and extraction ratios of Au(III) were 78.85% and 96.84%, the [BF_4_]^−^ concentrations in the aqueous phase were 0.122 mg/L and 18,040 mg/L, respectively. Although [HMIm]^+^[BF_4_]^−^ IL has a higher enrichment efficiency for Au(III), there was almost a 45% loss of the IL composed of [BF_4_]^−^ from the IL phase into the aqueous phase, leading to serious environmental pollution and difficult reuse of [HMIm]^+^[BF_4_]^−^. Meanwhile, there were only ultra-trace amounts of [BF_4_]^−^ found in the aqueous phase after the adsorption of Au(III) by immobilizing [HMIm]^+^[BF_4_]^−^ on UiO-66. Obviously, the loss of [BF_4_]^−^ was restrained because [BF_4_]^−^ anions were effectively confined in UiO-66 cages as we initially expected.

**Figure 4:**
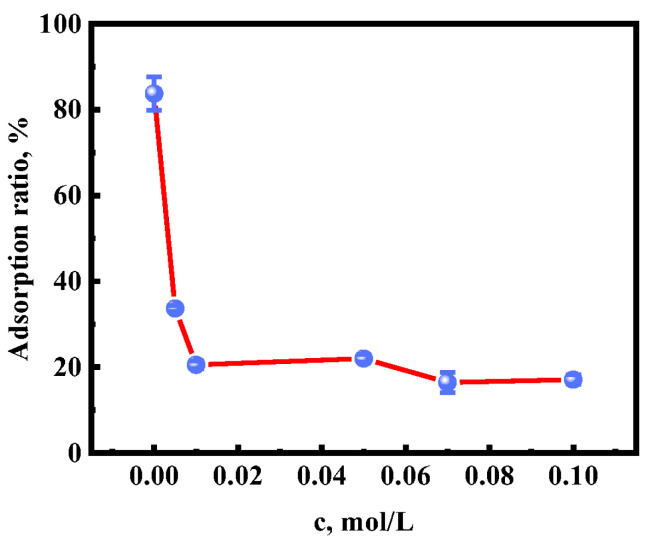
Effects of different concentrations of BF_4_^−^ on Au(III) adsorption (c _Au(III)_ = 60 mg/L, V _Au(III)_ = 20 mL, m _[HMIm]+[BF4]-@UiO-66_ = 10 mg, t = 6 h, T = 35 °C, pH = 2).

To further explore the stronger interaction between UiO-66 and the anions of [BF_4_]^−^ IL, the effect of [BF_4_]^−^ concentrations on Au(III) adsorption was investigated. As shown in Figure 4, the adsorption ratio of Au(III) decreased significantly from 83.72% to 17.10% with increasing [BF_4_]^−^ concentrations from 0 to 0.01 mol/L. The effect of [BF_4_]^−^ concentrations can be understood by considering the stronger interaction between the [BF_4_]^−^ anions and Zr^4+^ metal sites in UiO-66. The adsorbent preferred to adsorb [BF_4_]^−^ rather than [AuCl_4_]^−^, which was mainly affected by the anion radius. [BF_4_]^−^ with a smaller anionic radius (0.232 nm) is more conducive to transport into the pores of UiO-66. The increased amount of [BF_4_]^−^ in the adsorbent occupied adsorption sites and pore channels in UiO-66; thus, the adsorption of Au(III) on [HMIm]^+^[BF_4_]^−^@UiO-66 was significantly reduced.

Since the results confirm that [HMIm]^+^[BF_4_]^−^@UiO-66 adsorbed Au(III) certainly without ion exchange, to further explore the adsorption mechanism of Au(III), the effect of pH on adsorption was investigated, and the X-ray photoelectron spectroscopy (XPS) analyses of [HMIm]^+^[BF_4_]^−^@UiO-66 before and after adsorption were examined.

#### 2.3.2. Effect of pH

**Figure 5:**
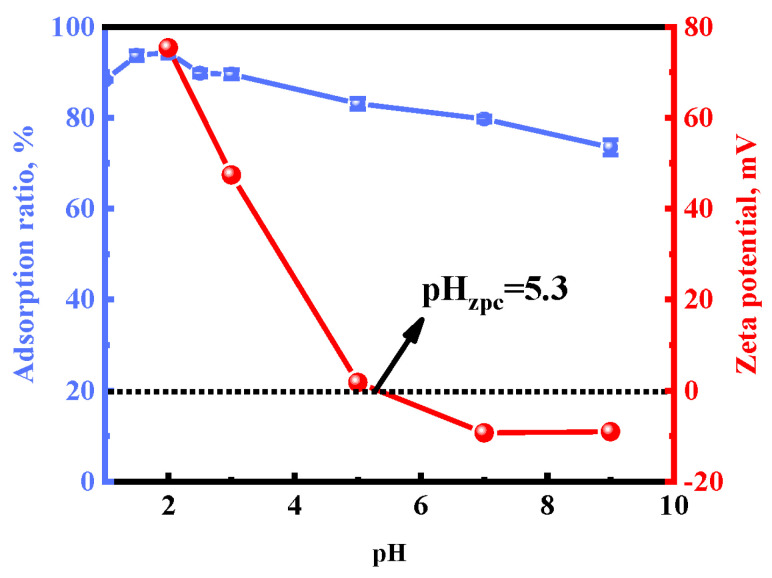
Effects of pH and zeta potential of [HMIm]^+^[BF_4_]^−^@UiO-66.

The pH effect on the surface charge of the adsorbent and metal ions is one of the fundamental factors affecting the adsorption rate. Here, the effect of pH varying from 1 to 9 on Au(III) adsorption was evaluated. The adsorption ratio of Au(III) was in the range of 88.28% to 94.37% when the pH was 1 to 2. Meanwhile, it decreased from 94.37% to 73.48% with increasing pH from 2 to 9. As far as we know, the species of Au(III) in solutions at pH 1–9 have negative charges, such as [AuCl_4_]^−^, [AuCl_3_(OH)]^−^, [AuCl_2_(OH)_2_]^−^, [AuCl(OH)_3_]^−^, and [Au(OH)_4_]^−^ [38,39,40]. As shown in Figure 5, the zero point charge (pH_zpc_) of [HMIm]^+^[BF_4_]^−^@UiO-66 is 5.3, indicating that the surface charge of [HMIm]^+^[BF_4_]^−^@UiO-66 is positively charged when the pH is lower than 5.3. The positively charged [HMIm]^+^[BF_4_]^−^@UiO-66 is conducive to the adsorption of the negatively charged Au(III) species. The decrease in the zeta potential of [HMIm]^+^[BF_4_]^−^@UiO-66 with the increase in the pH value is consistent with the lower pH value, facilitating Au(III) adsorption [45,46,47]. Thus, electrostatic interaction plays an important role in the adsorption process, and a pH of 2.0 was chosen in the following adsorption experiments. However, the adsorption ratio of Au(III) is 73.48% when the surface charge of [HMIm]^+^[BF_4_]^−^@UiO-66 is negatively charged at pH = 9, which indicates that [HMIm]^+^[BF_4_]^−^@UiO-66 has other attractions to Au(III).

#### 2.3.3. XPS analysis of [HMIm]^+^[BF_4_]^−^@UiO-66

**Figure 6:**
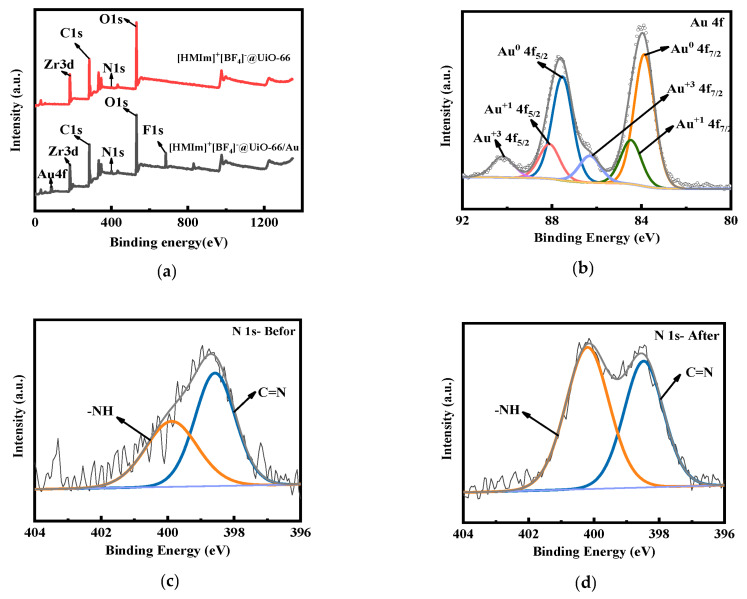
XPS spectra: (**a**) full spectra of [HMIm]^+^[BF_4_]^−^@UiO-66 and [HMIm]^+^[BF_4_]^−^@UiO-66/Au; (**b**) Au 4f spectra of [HMIm]^+^[BF_4_]^−^@UiO-66/Au; (**c**,**d**) N1s spectra of [HMIm]^+^[BF_4_]^−^@UiO-66 and [HMIm]^+^[BF_4_]^−^@UiO-66/Au.

To investigate the adsorption mechanism of Au(III), [HMIm]^+^[BF_4_]^−^@UiO-66 before and after the adsorption of Au(III) was analyzed via XPS. The complete XPS spectra of [HMIm]^+^[BF_4_]^−^@UiO-66 and [HMIm]^+^[BF_4_]^−^@UiO-66/Au are shown in Figure 6. Compared to the spectrum of [HMIm]^+^[BF_4_]^−^@UiO-66, a new Au4f peak was found for [HMIm]^+^[BF_4_]^−^@UiO-66/Au. This indicates that Au(III) was successfully adsorbed by this material. The peaks in the high-resolution Au4f spectrum of the gold adsorbent can be divided into Au4f 7/2 and Au4f 5/2, as shown in Figure 6b. The two peaks at 87.54 ev (Au4f 5/2) and 83.87 ev (Au4f 7/2) correspond to Au(0), while the two peaks at 88.12 ev (Au4f 5/2) and 84.47 ev (Au4f 7/2) correspond to Au(I) [25,45,46,48]. The results suggest that Au(III) was reduced to Au(0) and Au(I) by [HMIm]^+^[BF_4_]^−^@UiO-66, and that a redox mechanism exists during the adsorption process. The Au(0) area ratio is 64.68%, and the size of the peak area ratio indicates that gold mainly exists on the adsorbent in the form of Au(0) [46].

To understand the interactions between the gold and N atoms, the XPS N1s spectra of [HMIm]^+^[BF_4_]^−^@UiO-66 and [HMIm]^+^[BF_4_]^−^@UiO-66/Au were studied. In Figure 6c, the N1s spectra can be divided into C=N and -NH. The peak of the C=N binding energy changes from 398.57 ev to 398.47 ev, while the peak of the -NH binding energy changes from 399.88 ev to 400.2 ev, after adsorption. The peak area of C=N decreases while the peak area of NH increases relatively after adsorption. The results indicate that the electrons were transferred from N to Au(III). The N-containing functional groups were bound to Au(III) through complexation, and Au(III) was reduced to Au(I) and Au(0) [47,48]. The results prove the mechanism of Au(III) adsorption after IL immobilization, which not only avoids ion exchange in solvent extraction, but also provides a new adsorption site for Au(III).

In summary, the mechanism of the adsorption of Au(III) by [HMIm]^+^[BF_4_]^−^@UiO-66 has three parts: electrostatic interaction, coordination between Au(III) and N-containing functional groups, and a reduction of Au(III) to Au(I) and Au(0). The loss of [HMIm]^+^[BF_4_]^−^ IL in the aqueous phase caused by the miscibility of the water and anion exchange was effectively restrained because of the strong interaction between the IL and UiO-66.

### 2.4. Adsorption Kinetics

**Figure 7:**
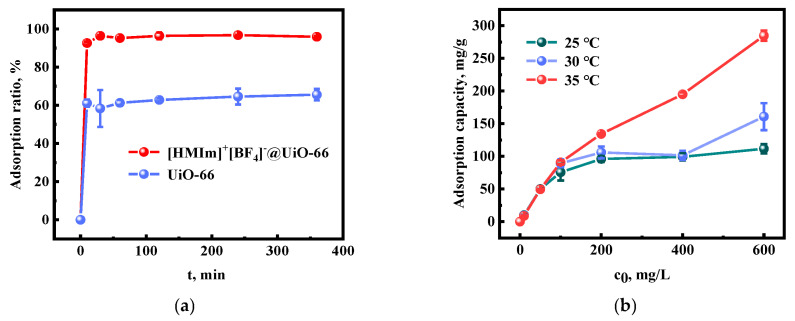
(**a**) Effect of adsorption time; (**b**) effect of the initial Au(III) concentration on the adsorption capacity of [HMIm]^+^[BF_4_]^−^@UiO-66.

The adsorption equilibrium time is one of the critical indicators for the evaluation of adsorbents. The effect of time on the adsorption of Au(III) by UiO-66 and [HMIm]^+^[BF_4_]^−^@UiO-66 was investigated. The results are shown in Figure 7a. It can be seen that the adsorption curve maintains the same trend. The adsorption of Au(III) by UiO-66 and [HMIm]^+^[BF_4_]^−^@UiO-66 increased rapidly from 0 to 10 min, reaching 61.09% and 92.60%, respectively, and as time increased, the adsorption ratio reached equilibrium at 50 and 25 min, respectively. Au(III) was rapidly adsorbed in the first 10 min. The adsorption ratio of Au(III) by [HMIm]^+^[BF_4_]^−^@UiO-66 was much higher than that of UiO-66. This indicates that the sequestration of the IL not only inhibited the exchange and decomposition of [BF_4_]^−^, but also provided a large number of adsorption sites for Au(III).

The data obtained were further fitted using pseudo-first-order (PFO, Equation (1)), pseudo-second-order (PSO, Equation (2)), and intraparticle diffusion (Id, Equation (3)) models to describe the adsorption behavior. The pseudo-first-order kinetic model assumes [49] that the adsorption process is physical adsorption, and its rate-limiting step is related to pore diffusion. The pseudo-second-order kinetic model assumes [39] that the adsorption process is chemical adsorption, and its rate-limiting step is a chemical reaction. The intraparticle diffusion model assumes [50] that the external mass transfer process leads to either rapid intraparticle diffusion or rate control steps.

(1) $$\ln\left(q_{e}-q_{t}\right)=\ln q_{e}-K_{1}t,$$

(2) $$\frac{t}{q_{t}}=\frac{1}{K_{2}q_{e}{}^{2}}+\frac{t}{q_{e}},$$

(3) $$q_{t}=K_{3}t^{1/2}+C,$$

where $q_{e}$ and $q_{t}$ (mg/g) are the metal amounts when adsorption equilibrium is reached and at time *t*, respectively, $K_{1}$ (1/min) is the pseudo-first-order rate constant, $K_{2}\;$(mg/g·min) is the pseudo-second-order rate constant, $K_{3}$(mg/g·min ^0.5^) is the particle diffusion rate constant, and C is the intercept, representing the thickness of the boundary layer.

**Figure 8:**
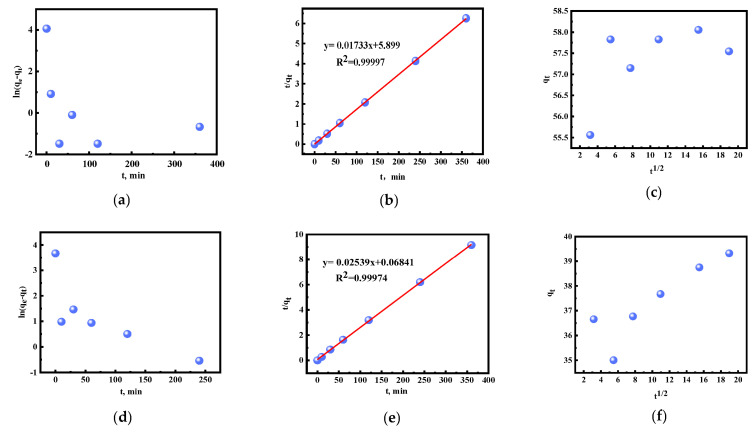
The fitting curves of UiO-66 and [HMIm]^+^[BF_4_]^−^@UiO-66: (**a**–**c**) UiO-66; (**d**–**f**) [HMIm]^+^[BF_4_]^−^@UiO-66; (**a**,**d**) pseudo-first-order; (**b**,**e**) pseudo-second-order; and (**c**,**f**) particle diffusion.

The experimental data were analyzed using pseudo-first-order, pseudo-second-order, and intraparticle diffusion models, and the parameters obtained are shown in Figure 8 and Table 1. For UiO-66 and [HMIm]^+^[BF_4_]^−^@UiO-66, the pseudo-second-order model has the highest correlation coefficient (R^2^_UiO-66_ **=** 0.9998 and R^2^_[HMIm]+[BF4]-@UiO-66_ **=** 0.9934), meaning it has a good linear correlation. In addition, the experimental values of Q in the PSO kinetics are closer to the theoretical values, which further demonstrates that the adsorption process of Au(III) can be better reflected by the PSO kinetic model. This suggests that the rate-limiting step of the Au(III) adsorption behavior of UiO-66 and [HMIm]^+^[BF_4_]^−^@UiO-66 is a chemical reaction. In contrast, the adsorption capacity of [HMIm]^+^[BF_4_]^−^@UiO-66 is positively correlated with the number of adsorption sites [51,52,53].

### 2.5. Isotherm Study

The adsorption isotherm is an important indicator to evaluate the maximum adsorption capacity of the adsorbent. The variation in the adsorption capacity with the initial Au(III) concentration at three temperatures (25, 30, and 35 °C) is shown in Figure 7b. Furthermore, the adsorption capacity increases with increasing initial Au(III) concentration. It can be adsorbed entirely at low concentrations because a sufficient number of adsorption sites are provided by the adsorbent. Due to the limited number of adsorption sites, the adsorption capacity tends to increase gradually and slowly with increasing Au(III) concentration, which leads to adsorption saturation. The obtained data were fitted and analyzed with the Langmuir model (Equation (4)), Freundlich model (Equation (5)), and Dubinin–Radushkevich (D–R) model (Equation (6)). The Langmuir model assumes [54] that adsorption is monolayer adsorption on a uniform surface, and that the adsorption and desorption are in dynamic equilibrium. The Freundlich isotherm model assumes [55] that adsorption is multilayer adsorption and occurs on heterogeneous surfaces. The D–R isotherm model assumes [56] that the adsorption process is not layer-by-layer adsorption on the adsorbent surface but related to the micropore volume.

(4) $$\frac{c_{e}}{q_{e}}=\frac{1}{K_{L}q_{m}}+\frac{c_{e}}{q_{m}},$$

(5) $$\ln q_{e}=\ln K_{F}+\frac{1}{n}c_{e},$$

(6) $$\ln q_{e}=\ln q_{m}-\beta \varepsilon^{2},\;\varepsilon =\mathrm{RT}\ln\left(1+\frac{1}{c_{e}}\right),$$

where $K_{L}$(L/mg) is the Langmuir constant related to the affinity of the binding site, $K_{F}$((mg/g)/(L/mg)^1/n^) shows the Freundlich constant associated with the adsorption strength, $q_{m}$ (mg/g) and n express the highest adsorption capacity and the coefficient of the Freundlich model, respectively, β represents the D–R isotherm constant, R represents the universal gas constant (8.314 J/mol·K), and T represents the temperature (K).

The separation factor (*R_L_*) describes the basic characteristics and feasibility of the Langmuir isotherm:

(7) $$R_{L}=\frac{1}{1+K_{L}c_{0}}$$

**Figure 9:**
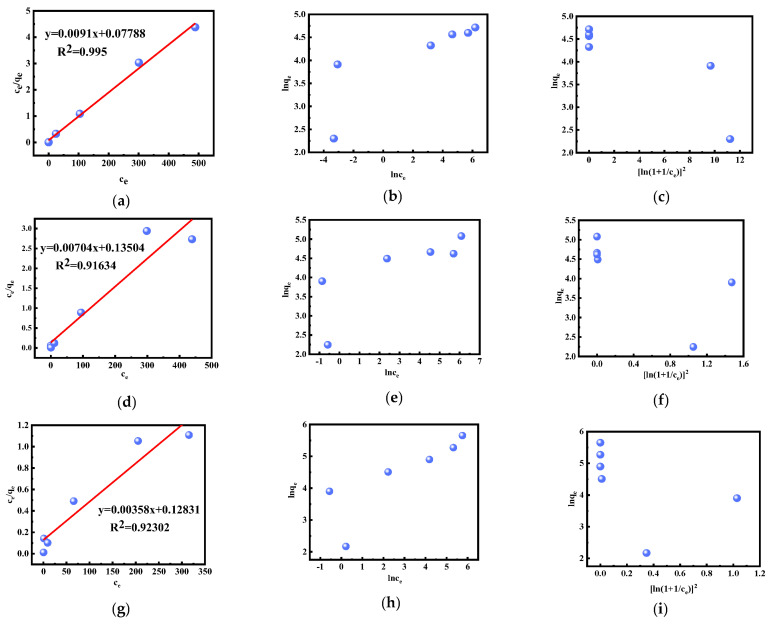
Isotherm fitting: (**a**) Langmuir model at 25 °C; (**b**) Freundlich model at 25 °C; (**c**) D–R model at 25 °C; (**d**) Langmuir model at 30 °C; (**e**) Freundlich model at 30 °C; (**f**) D–R model at 30 °C; (**g**) Langmuir model at 35 °C; (**h**) Freundlich model at 35 °C; and (**i**) D–R model at 35 °C.

The experimental results were analyzed using the Langmuir, Freundlich, and D–R models, and the parameters obtained are shown in Figure 9a–i and Table 2. The results show that the adsorption isotherms of Au(III) were more consistent with the Langmuir model (R^2^ = 0.995, 0.91634, 0.92302), and the theoretical maximum adsorption amounts of Au(III) at the three temperatures were 109.89, 142.05, and 279.33 mg/g, which are very close to the actual maximum adsorption amounts of 111.57, 160.77, and 284.64 mg/g, indicating that the adsorption of Au(III) is monolayer adsorption at a specific homogeneous location on the adsorbent surface [47,48,57]. Moreover, the *R_L_* values of Au(III) in the Langmuir model were all below 1, which indicates that the adsorption is appropriate [53]. In addition, the maximum adsorption of [HMIm]^+^[BF_4_]^−^@UiO-66 (284.64 mg/g) was higher than that of UiO-66 (203.42 mg/g) (see SI for detailed results).

### 2.6. Thermodynamic Study

The influence of the adsorption temperature on the adsorption process is significant and explains the adsorption thermodynamics with relevant thermodynamic parameters. The data obtained were evaluated with the following equations (Equations (8)–(10)) containing classical thermodynamic parameters [25]:

(8) $$K_{c}=\frac{q_{e}}{c_{e}},$$

(9) $$\Delta G=-\mathrm{RT}\ln K_{c},$$

(10) $$\ln K_{c}=\frac{\Delta S}{R}-\frac{\Delta H}{\mathrm{RT}},$$

where $K_{c}$, T (K), and R (8.314 J/mol·K) are the thermodynamic equilibrium constant, the adsorption temperature, and the gas constant, respectively, ΔS (J/mol/K), ΔH (KJ/mol), and Δ*G* (KJ/mol), respectively, are the changes in the entropy, enthalpy, and Gibb’s free energy.

**Figure 10:**
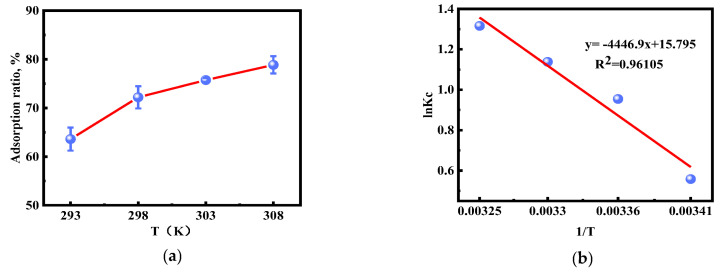
(**a**) Effect of temperature; (**b**) plot of ln Kc versus 1/T.

As shown in Figure 10, the Au(III) adsorption of [HMIm]^+^[BF_4_]^−^@UiO-66 was enhanced with increasing temperature. The thermodynamic parameters at different temperatures are summarized in Table 3. The increase in temperature favors the increase in the number of active molecules. Thus, the adsorption of Au(III) by the adsorbent is promoted, indicating that the adsorption process is heat absorption. It was found that Δ*G* was negative at different temperature conditions, indicating that the reaction process is spontaneous and feasible [20,58]. Additionally, the negative value of Δ*G* was increased with increasing temperature, indicating that the higher the temperature, the more spontaneous and favorable the adsorption of Au(III). The positive value of ΔH indicates that the adsorption is a heat-absorbing reaction. On the contrary, a positive value of ΔS indicates that the system’s degrees of freedom and disorder are increased, which favors an increase in the frequency of collisions between Au(III) and the adsorbent [42,45,48,59].

### 2.7. Selectivity and Practical Application

**Figure 11:**
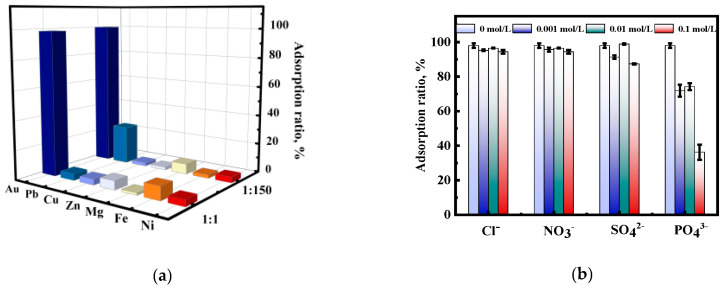
(**a**) Effect of coexisting metal ions on Au(III) adsorption (c _Au(III) and coexisting ions_ = 100 mg/L, c _Au(III)_ = 10 mg/L and c _coexisting ions_ = 1500 mg/L); (**b**) effect of acid radical ions on Au(III) adsorption.

E-waste containing Au(III) coexists with other metal ions; therefore, the adsorption selectivity of Au(III) was studied to better evaluate the adsorbent’s performance. Mg(II), Cu(II), Zn(II), Pb(II), Fe(II), and Ni(II) were chosen as background ions to study the selectivity of [HMIm]^+^[BF_4_]^−^@UiO-66. As shown in Figure 11a, when the concentration ratio of Au(III) to other coexisting ions was 1:1, Au(III) adsorption on the adsorbent reached 98.5%. In contrast, almost no other metal ions were adsorbed. Considering that the concentration of coexisting ions in e-waste leachate is several times higher than that of Au(III), the adsorption of Au(III) with other coexisting ions at a concentration ratio of 1:150 was investigated. The results show that the adsorption of Au(III) remained unaffected by the high concentration of coexisting ions, and that Au(III) could be 100% adsorbed by the adsorbent. The excellent Au(III) adsorption performance of the adsorbent can be attributed to the physicochemical properties of the metal atoms, such as the ionic radius (R), electronegativity (Xm), and covalent index (Xm^2^r). The Xm^2^r (5.48) and Xm (2.54) of Au(III) are higher, which allows Au(III) to be preferentially adsorbed [46]. Additionally, according to hard–soft acid–base (HSAB) theory, Au(III) can form strong bonds with N-containing functional groups, which may also contribute to its high adsorption [46,60]. In addition, at a lower pH, other coexisting ions may exist as cations or neutrals. Therefore, the coexisting ions are not adsorbed by [HMIm]^+^[BF_4_]^−^@UiO-66 due to electrostatic repulsion with positively charged [HMIm]^+^[BF_4_]^−^@UiO-66 on the surface [61]. In addition, some anions will be inevitably introduced into the system during the leaching of Au(III) from e-waste. Therefore, the effect of several representative anions (Cl^−^, SO_4_^2−^, PO_4_^3−^, and NO_3_^−^) on Au(III) adsorption at different concentrations (0, 0.001, 0.01, and 0.1 mol/L) was investigated. As can be seen in Figure 11b, the adsorption of Au(III) was inhibited to a greater extent by PO_4_^3−^ as the anion concentration increased. When PO_4_^3−^ was 0.1 mol/L, the adsorption of Au(III) was only 36%. On the contrary, the adsorption of Au(III) was slightly inhibited by SO_4_^2−^. When SO_4_^2−^ was 0.1 mol/L, the adsorption of Au(III) was still above 85%, while Cl^−^ and NO_3_^−^ hardly affected the adsorption of Au(III). In the presence of different Cl^−^ and NO_3_^−^ concentrations, Au(III) adsorption remained at around 95%. Therefore, leaching agents containing Cl^−^ and NO_3_^−^ media are preferred in the Au(III) leaching process. When the commonly used aqua regia ablates e-waste to extract Au(III), the adsorption of Au(III) on the composites is not affected. In addition, the aqua regia-based leaching of e-waste is a flexible and low-cost method. At present, it is also a widespread process in the industry [62,63].

**Figure 12:**
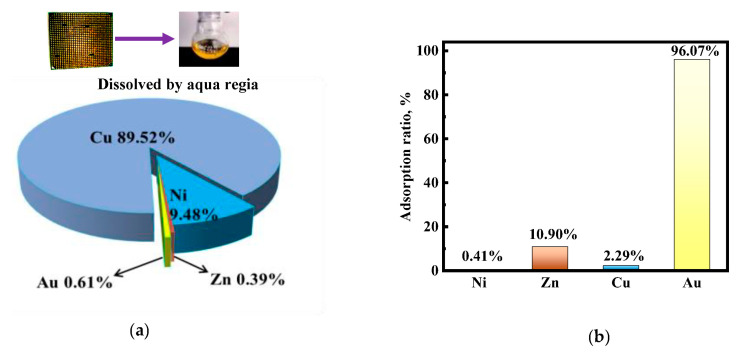
(**a**) Percentage of major metal elements; (**b**) adsorption rate of each metal ion.

The practical application value of [HMIm]^+^[BF_4_]^−^@UiO-66 was further evaluated to recover precious metals from discarded CPU motherboard pins. The main metal elements in CPU pins are shown in Figure 12a, and the adsorption rate of each metal ion is shown in Figure 12b. In the figure, it can be seen that the total percentage of the primary metals Ni(II), Cu(II), and Zn(II) in the CPU is more than 99%. In the presence of high concentrations of coexisting ions, it is clear that Ni(II), Cu(II), and Zn(II) are hardly adsorbed. In contrast, up to 96% of Au(III) is adsorbed on the adsorbent at low concentrations. The high selectivity of [HMIm]^+^[BF_4_]^−^@UiO-66 for Au(III) in practical applications is consistent with the results of previous selectivity studies, further demonstrating that [HMIm]^+^[BF_4_]^−^@UiO-66 has practical application value.

### 2.8. Reusability

**Figure 13:**
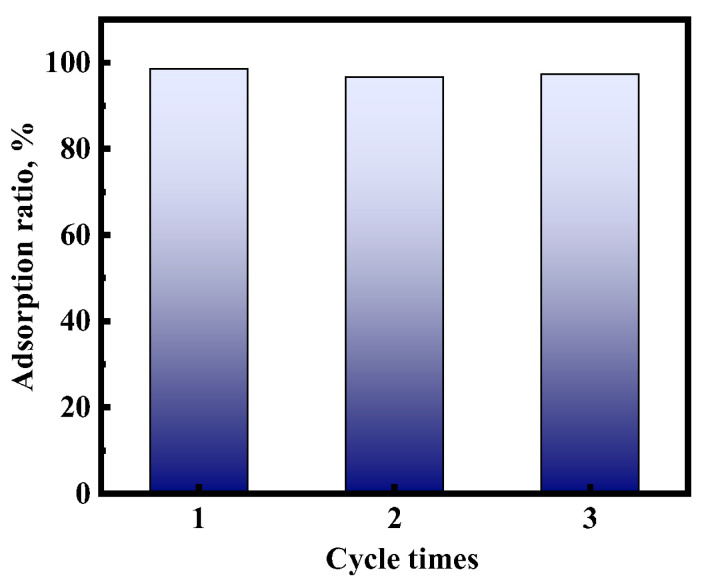
Reusability of [HMIm]^+^[BF_4_]^−^@UiO-66.

In order to understand the recyclability of the adsorbent and to judge whether it has practical value, the reusability of the composite was investigated. The Au(III) adsorption rate of [HMIm]^+^[BF_4_]^−^@UiO-66 in the reusability experiments is shown in Figure 13. After three consecutive cycles, the removal of Au(III) was greater than 95%, and there was no obvious decrease. However, no subsequent experiments were performed due to the large material loss during adsorption resolution. The results show that [HMIm]^+^[BF_4_]^−^@UiO-66 has relatively stable adsorption properties and can be used as an excellent adsorbent for separating Au(III) from aqueous media.

## 3. Discussion

[HMIm]^+^[BF_4_]^−^@UiO-66 was prepared as an adsorbent material for Au(III) recovery from acidic solutions, and it effectively avoided a series of contamination problems associated with the dissolution of ILs in water due to ion exchange. A series of characterizations, including SEM, FTIR, XRD, and N_2_ adsorption and desorption experiments, confirmed the successful sequestration of ILs, and the maximum adsorption of Au(III) by [HMIm]^+^[BF_4_]^−^@UiO-66 at pH 2.0 and 35 °C was 284.64 mg/g. In the kinetic and thermodynamic studies, the adsorption process was found to be an endothermic, feasible, and spontaneous reaction, while the rate-limiting step of adsorption was found to be a chemical reaction. In the isotherm studies, the adsorption processes at different temperatures were consistent with the Langmuir model. [HMIm]^+^[BF_4_]^−^@UiO-66 showed good selectivity for Au(III) adsorption and successfully recovered Au(III) from e-waste. The effect of the pH on Au(III) adsorption and the XPS results indicated that the Au(III) adsorption mechanism was either an electrostatic, redox, coordination, or complexation mechanism. The results indicate that [HMIm]^+^[BF_4_]^−^@UiO-66 successfully enclosed ILs. As an advanced adsorptive material for gold recovery from e-waste, [HMIm]^+^[BF_4_]^−^@UiO-66 can be reused for three cycles without any significant decrease in the adsorption rate. It has a simple synthesis, appropriate adsorption kinetics and adsorption capacity, and excellent selectivity and regeneration, and it can successfully recover gold in practical applications. The results of the present work show that the material has application value and practicability in industry.

## 4. Materials and Methods

### 4.1. Materials and Chemicals

The standard stock solution of Au(III) (1000 mg/L) was obtained from the Shandong Metallurgical Research Institute. The working solutions were prepared daily by diluting the standard stock solution with deionized water. Zirconium chloride (ZrCl_2_, 99.5%) and terephthalic acid (TPA, 99.0%) were obtained from Shanghai McLean Biochemical Technology Co., Ltd, Shanghai, China. All of the ILs, including 1-ethyl-3-methylimidazole tetrafluoroborate ([EMIm]^+^[BF_4_]^−^, ≥98%), 1-octyl-3-methylimidazole tetrafluoroborate ([OMIm]^+^[BF_4_]^−^, ≥98%), 1-butyl-3-methylimidazole tetrafluoroborate ([BMIm]^+^[BF_4_]^−^, ≥98%), 1-hexyl-3-methylimidazole tetrafluoroborate ([HMIm]^+^[BF_4_]^−^, ≥98%), 1-hexyl-3-methylimidazole hexafluorophosphate ([HMIm]^+^[BF_6_]^−^, 99%), 1-hexyl-3-methylimidazole bis trifluoromethylsulfonimide salt ([HMIm]^+^[NTf_2_]^−^, 99%), and 1-hexyl-3-methylimidazole acetate ([HMIm]^+^[OAc]^−^, ≥98%) were provided by the Lanzhou Institute of Chemical Physics, Chinese Academy of Sciences. Other chemicals were of analytical grade and obtained from Sinopharm Chemical Reagent limited corporation. All reagents and solvents were used without additional purification.

### 4.2. Synthesis of Adsorbents

#### 4.2.1. Synthesis of UiO-66

The synthesis process of UiO-66 was based on a previously reported method [64]. The details are reported in the Supplementary Materials.

#### 4.2.2. Synthesis of IL/UiO-66

Amounts of 1.5 g UiO-66, 1 g ILs, and 3 mL C_2_H_5_OH were mixed in a glass bottle and stirred at room temperature for 15 h. Then, the composite was filtered, washed with C_2_H_5_OH, and dried in a vacuum oven overnight at 80 °C.

### 4.3. Characterization

The remaining contents of Au(III) in water and other metal ions (Mg(II), Cu(II), Zn(II), Pb(II), Fe(III), and Ni (II)) were determined using a flame atomic absorption spectrometer (TAS990, Beijing Purkinje General Instrument Co., Ltd., Beijing, China) (for specific experimental details, see the SI). The concentration of [BF_4_]^−^ in the aqueous phase was determined using a high-pressure ion chromatograph (Integrion, Thermo Fisher Scientific, Massachusetts, US) with an ion Dionex IonPac AS 11-HC chromatography column with 20 mM KOH mobile phase at a flow rate of 1.0 mL/min (the sample injection volume was 25 μL, and the column oven temperature was 30 °C). The FT-IR spectra of UiO-66 and [HMIm]^+^[BF_4_]^−^@UiO-66 were investigated in the range of 400–4000 cm^−1^ via Fourier transform infrared spectroscopy (FT-IR) (IRAffinity-15, Shimadzu production Institute, Kyodo, Japan) with KBr pellets. The morphologies of UiO-66 and [HMIm]^+^[BF_4_]^−^@UiO-66 were recorded using a scanning transmission electron microscope (SEM) (STEM, FEI Tecnai G2 TF20, Frequency Electronics, Inc. Hillsboro, US). The zeta potential of [HMIm]^+^[BF_4_]^−^@UiO-66 was measured using a nanometer particle size and zeta potential analyzer (Malvern Nano ZS, Malvern, UK). The powder X-ray diffraction spectrometry (PXRD) patterns of UiO-66 and [HMIm]^+^[BF_4_]^−^@UiO-66 were captured by a D/max-2500 diffractometer (Rigaku, Tokyo, Japan) using CuKα radiation (λ = 1.5418 Å). The Brunauer–Emmett–Teller (BET) surface area and pore size distribution were calculated using N_2_ adsorption–desorption methods with an ASAP 2020 V4.00 G instrument (Micromeritics Instrument Crop., Norcross, GA, USA) at 77 K. The X-ray photoelectron spectroscopy (XPS) analysis was conducted using a ThermoFisher scientific spectrometer (Nexsa base, Thermo Fisher Scientific, Massachusetts, US) equipped with a micro-focused monochromatic Al Kα source (hν = 1486.6 eV).

### 4.4. Batch Adsorption Experiment

The batch adsorption experiments were performed to study the Au(III) adsorption performance of [HMIm]^+^[BF_4_]^−^@UiO-66. Generally, 10 mg [HMIm]^+^[BF_4_]^−^@UiO-66 was added into a 50 mL plastic centrifugal tube containing 10 mL Au(III) solution with different concentrations at pH 2. The solution was shaken in a constant oscillator at 170 rpm for the desired duration, and then the mixture was filtered through a 0.45 μm filter membrane to separate the adsorbent from the aqueous phase.

Adsorption experiments were performed under optimized parameters (oscillation frequency: 170 rpm; t: 6 h; pH: 2; T: 35°C; V _Au(III)_: 10 mL) unless otherwise indicated. The effect of pH was studied in the pH range of 1–9 (adjusted by adding diluted NaOH or HCl solutions). The experiment of the adsorption kinetics was carried out at different adsorption times ranging from 0 to 360 min. In contrast, the experiment of the adsorption isotherms was evaluated at different initial Au(III) concentrations ranging from 0 to 600 mg/L. The thermodynamic experiment was studied by controlling the temperature (from 298 to 308 K). The selectivity of the adsorbent for Au(III) was studied with different concentrations of Au(III) in the hybrid solution prepared by dissolving HAuCl_4_, MgCl_2_, CuCl_2_, ZnCl_2_, PbCl_2_, FeCl_3_, and NiCl_2_ in DI water. With initial Au(III) concentrations of 10 and 100 mg/L, the mass ratios of Au(III) to coexisting ions were 1:150 and 1:1, respectively. The effect of anions was examined in the presence of Cl^−^, SO_4_^2−^, PO_4_^3−^, and NO_3_^−^, and the concentrations were set to 0, 0.001, 0.01, and 0.1 mol/L.

Liquid–liquid extraction experiments were performed by adding 1 mL [HMIm]^+^[BF_4_]^−^ to 10 mL of 150 mg/L Au(III) solution for 6 h with shaking under optimized parameters. After centrifugation (3000 r/min) of the mixed solution for 5 min, the aqueous was taken for measurement.

The regeneration experiment of [HMIm]^+^[BF_4_]^−^@UiO-66 was implemented as follows: 20 mg [HMIm]^+^[BF_4_]^−^@UiO-66 was mixed to 20 mL Au(III) aqueous solution (60 mg/L) at pH 2. Then, the mixed solution was shaken for 6 h at 35 °C and was separated by high-speed centrifugation (8000 r/min). The supernatant was tested to obtain the remaining Au(III) concentration. The residual solid was immersed for about 12 h with 20 mL 1 mol/L HCl and 5% thiourea solution (the gold was eluted into an acid thiourea solution), rinsed three times with DI water, and executed to the second time of adsorption–desorption cycle. The adsorption capacity and ratio of Au(III) were calculated using the following equations:

(11) $$q=\frac{\left(c_{0}-c_{e}\right)}{m}V,$$

(12) $$\mathrm{Adsorption}\;\mathrm{ratio}=\frac{c_{0}-c_{e}}{c_{0}}\times 100\%\;,$$

where *q* (mg/g) is the Au(III) adsorption capacity, $c_{0}$ and $c_{e}$ (mg/L) are the initial and equilibrium concentrations of Au(III) in solution, respectively, *m* (mg) is the mass of the adsorbent used, and *V* (mL) is the volume of the Au(III) solution.

### 4.5. Recovery of Au(III) from Waste CPUs

The waste CPUs we used had an array of pins. The pins were detached from the CPU and immersed in aqua regia solution for 2 h (magnetically stirred for 1 h at room temperature and then 1 h at 75 °C) until the pins were wholly dissolved without residue. The obtained solution was diluted 10 times with DI water. Then, 10 mL of the diluted solution and 10 mg of adsorbent were added into a centrifuge tube, shaken for 6 h, and then filtered through a filter membrane.

## Figures and Tables

**Table 1:** The kinetic parameters of pseudo-second-order model.

	Pseudo-Second-Order		
	R^2^	qe(fit)	Qe(exp)
UiO-66	0.9998	39.326	39.541
[HMIm]^+^[BF_4_]^−^@UiO-66	0.9934	64.185	59.844

**Table 2:** The parameters of the Langmuir model.

	Langmuir		
	K_L_	q_m_	R^2^
25 °C	0.11685	109.89	0.995
30 °C	0.05213	142.05	0.91634
35 °C	0.02790	279.33	0.92302

**Table 3:** The thermodynamic parameters at different temperatures.

T(K)	*Kc*	Δ*G* (kJ/moL)	ΔH (kJ/moL)	ΔS (kJ/moL)
293	1.7473	−1.3594		
298	2.5965	−2.3641	36.971	0.13132
303	3.1197	−2.8662		
308	3.7279	−3.3695		

## Data Availability

Data are contained within the article or Supplementary Materials.

## Supplementary Materials

The following supporting information can be downloaded at: https://www.mdpi.com/article/10.3390/molecules28052165/s1.

## Sample Availability

Samples of [HMIm]^+^[BF_4_]^−^@UiO-66 are available from the authors.
