# Supplementary material for: Extraction of Gold Based on Ionic Liquid Immobilized in UiO-66: An Efficient and Reusable Way to Avoid IL Loss Caused by Ion Exchange in Solvent Extraction
Source: Molecules. 2023 Feb 25;28(5):2165. doi: 10.3390/molecules28052165 (PMC10004778; doi:10.3390/molecules28052165)
Supplement: Supplementary file 1 [file molecules-28-02165-s001.zip › molecules-2223150-supplementary.pdf]

Supplementary Information

1. Synthesis of UiO–66

1.165 g $ZrCl_4$, 0.831 g TPA, 0.875 mL of HCl, and 29.3 mL of DMF were mixed in a teflon–lined stainless steel autoclave. The mixture was heated at 180℃ for 24 h. The resultant UiO–66 was further purified by mixing UiO–66 with DMF (1 g UiO–66 to 50 mL DMF) in a teflon–lined stainless steel autoclave, and the mixture was heated at 150℃ for 5 h and then filtered and rinsed with distilled water. Finally, the obtained UiO–66 were oven–dried at 150℃ for 12 h.

2. SEM image of UiO–66 and $[HMIm]^+[BF_4]^-$@UiO–66

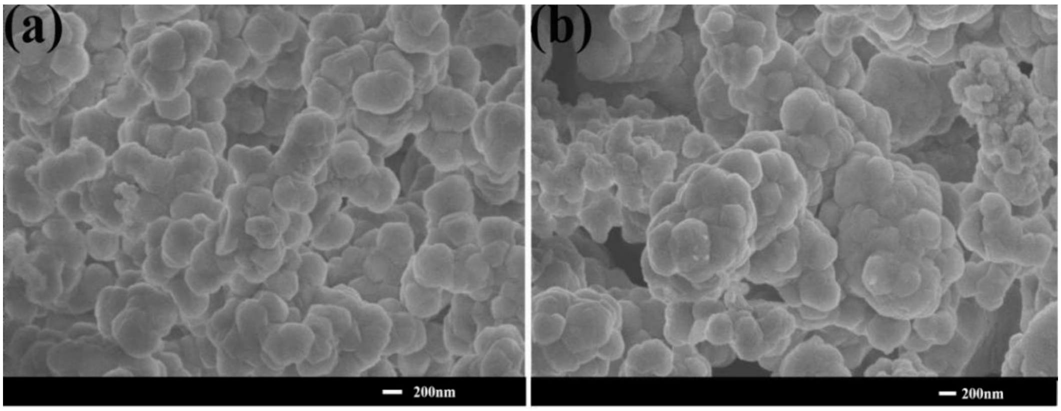

Supplementary Figure S1. (**a**) SEM image of UiO–66; (**b**) SEM image of $[HMIm]^+[BF_4]^-$@UiO–66.

3. BF_4^- ion chromatogram

3.1 BF_4^- ion chromatogram in water under different conditions

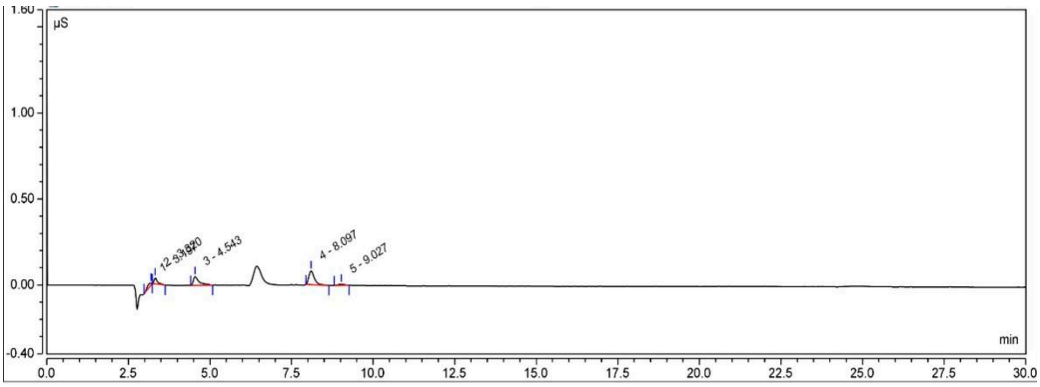

Supplementary Figure S2. BF_4^- ion chromatogram: (**a**) DI water.

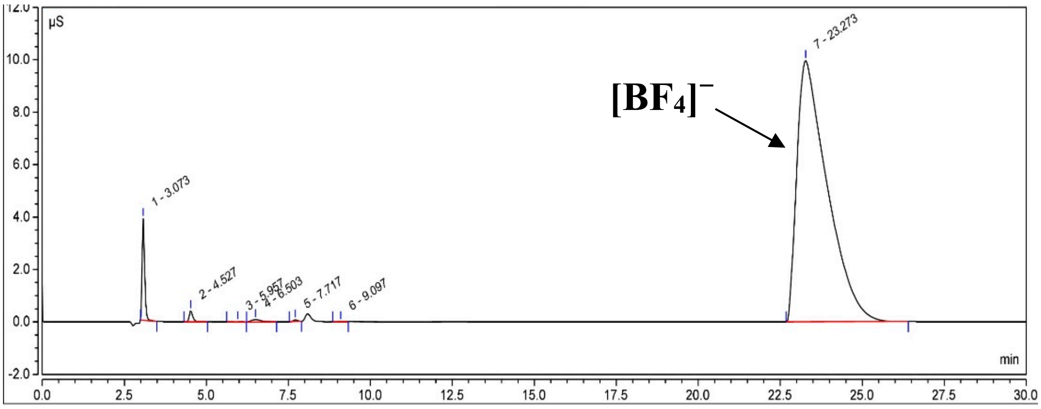

(**b**) $NaBF_4$ (100 mg/L).

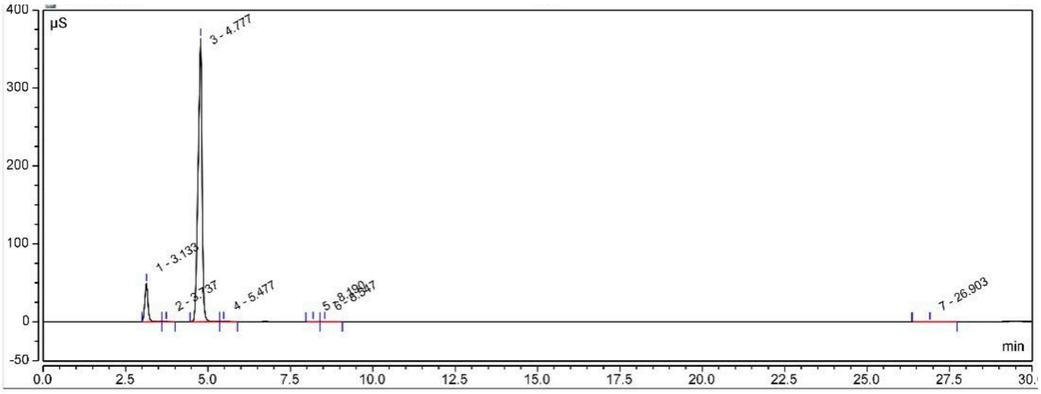

(**c**) The supernatant after adsorption of the 0 mg/L Au(III) solutions on the $[HMIm]^+[BF_4]^-$@UiO−66 at pH 2 ($m_{[HMIm]+[BF4]-@UiO-66}$=10 mg, $V_{Au(III)}$ = 10 mL, T = 35℃, t = 6 h).

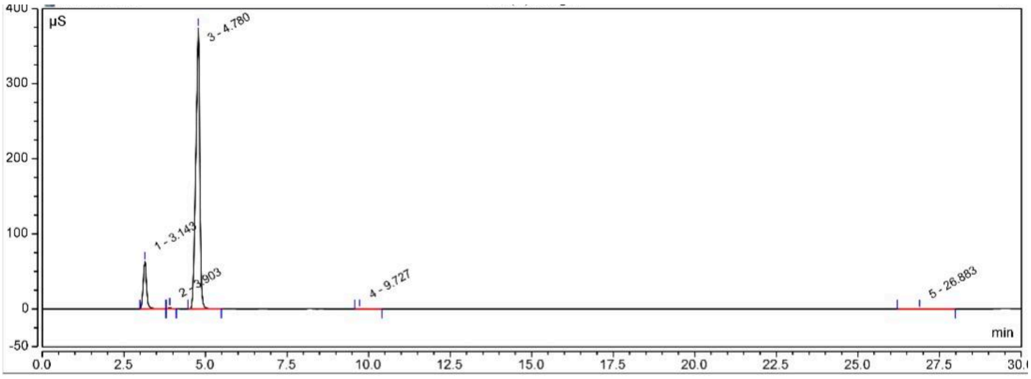

(**d**) The supernatant after adsorption of the 150 mg/L Au(III) solutions on the $[HMIm]^+[BF_4]^-$@UiO−66 at pH 2 ($m_{[HMIm]+[BF4]-@UiO-66}$=10 mg, $V_{Au(III)}$ = 10 mL, T = 35℃, t = 6 h).

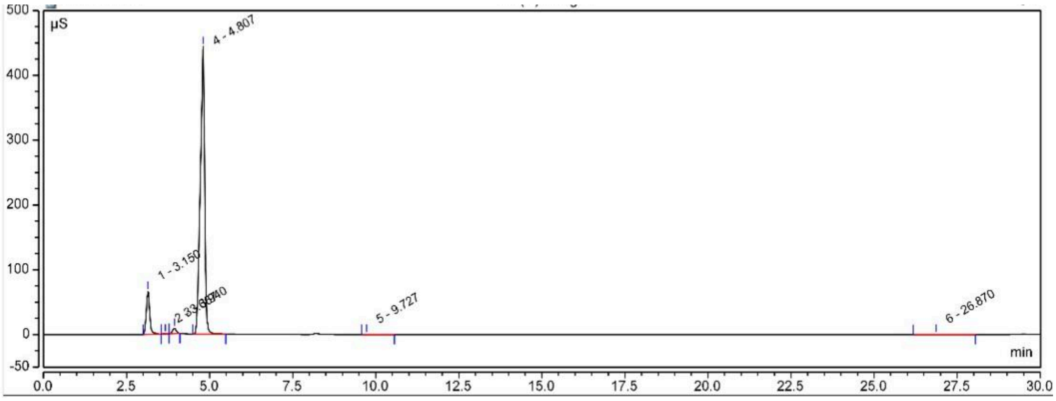

(**e**) The supernatant after adsorption of the 300 mg/L Au(III) solutions on the $[HMIm]^+[BF_4]^-$@UiO−66 at pH 2 (m $_{[HMIm]+[BF4]−@UiO−66}$=10 mg, V $_{Au(III)}$ = 10 mL, T = 35℃, t = 6 h).

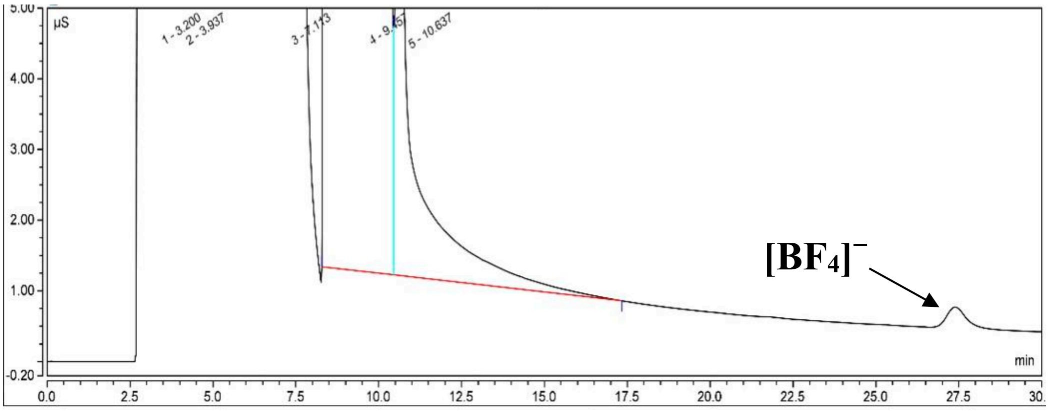

(**f**) The supernatant after adsorption of the 150 mg/L Au(III) solutions on the $[HMIm]^+[BF_4]^-$@UiO−66 at aqua regia after diluted 10 times (HCl： 1.2 mol/L, and HNO_3: 1.6mol/L) (m$_{[HMIm]+[BF4]−@UiO−66}$=10 mg, V $_{Au(III)}$ =10 mL, T = 35℃, t = 6 h).

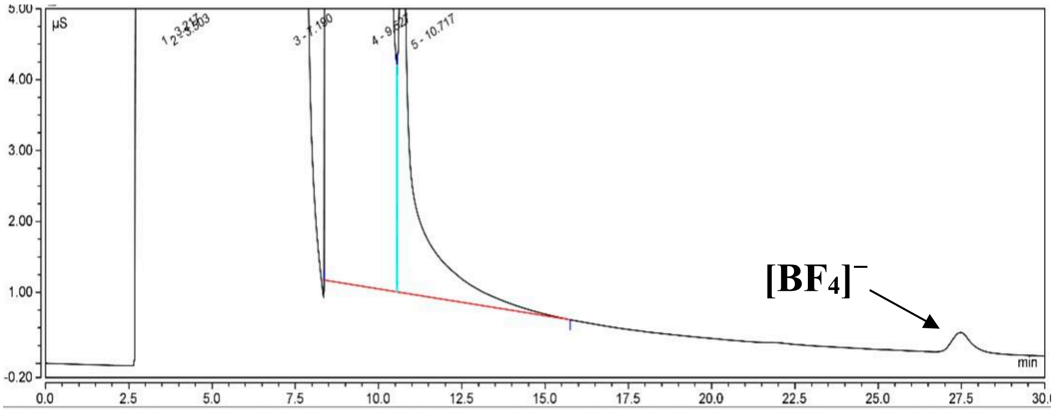

(**g**) The supernatant after adsorption of the 300 mg/L Au(III) solutions on the $[HMIm]^+[BF_4]^-$@UiO−66 at aqua regia after diluted 10 times (HCl: 1.2 mol/L, and HNO_3: 1.6 mol/L) (m$_{[HMIm]+[BF4]−@UiO−66}$=10 mg, V $_{Au(III)}$ =10 mL, T = 35℃, t = 6 h).

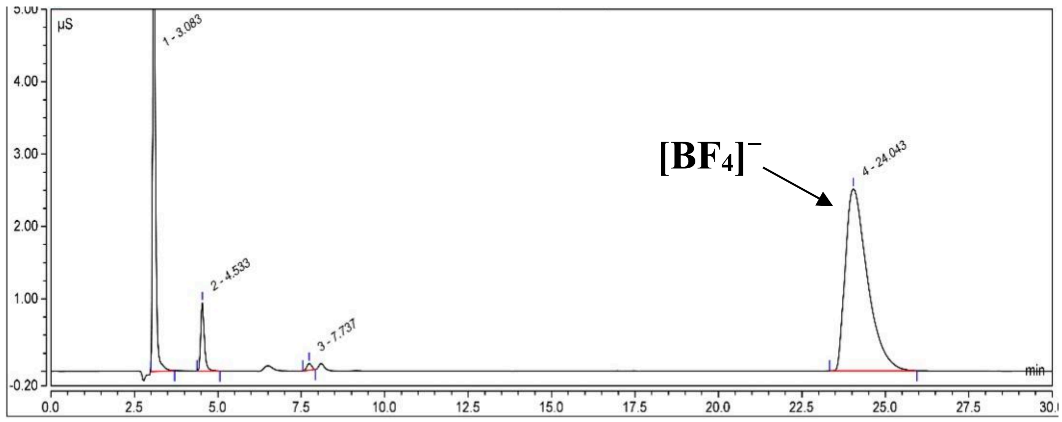

(**h**) The supernatant after extraction of the 150 mg/L Au(III) solutions from $[HMIm]^+[BF_4]^-$ (measured after dilution 1000) at pH 2 ($V_{[HMIm]+[BF4]-}$=1 mL, $V_{Au(III)}$ = 10 mL, T = 35℃, t = 6 h).

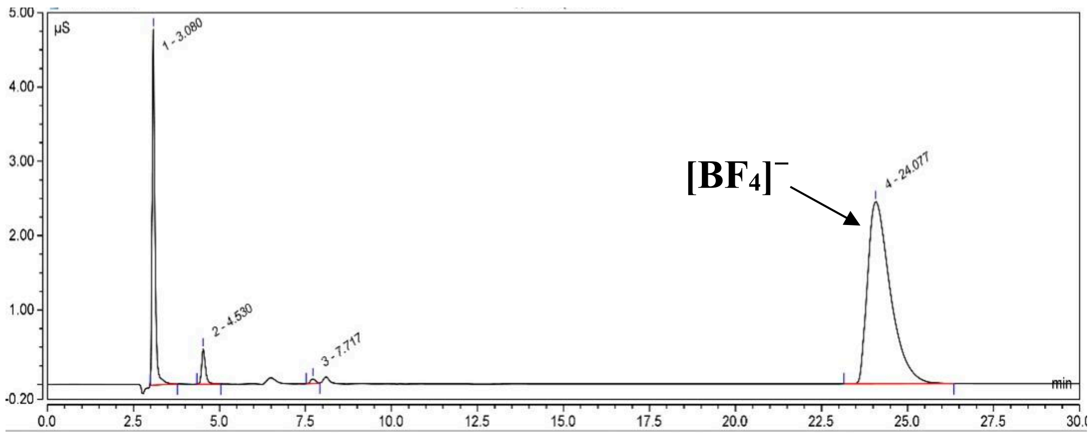

(**i**). The supernatant after extraction of the 300 mg/L Au(III) solutions from $[HMIm]^+[BF_4]^-$ (measured after dilution 1000) at pH 2 ($V_{[HMIm]+[BF4]-}$=1 mL, $V_{Au(III)}$ = 10 mL, T = 35℃, t = 6 h)

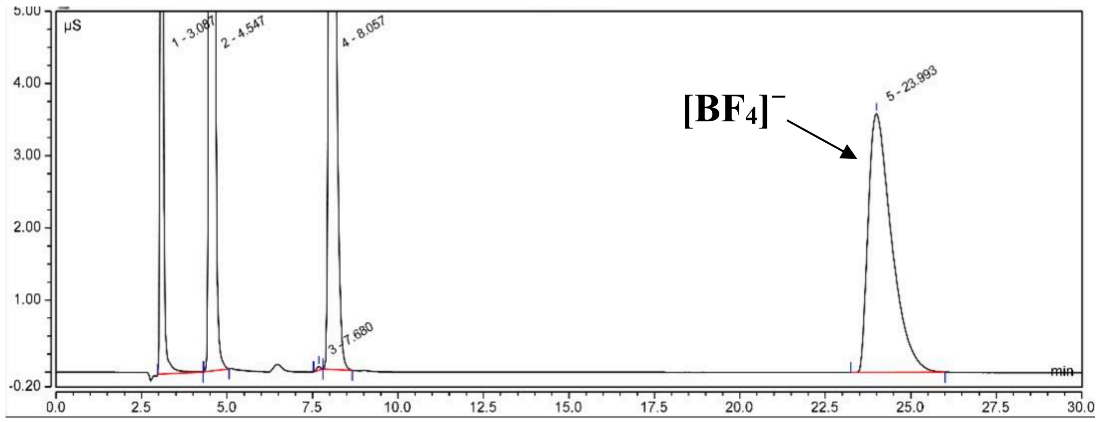

(**j**) The supernatant after extraction of the 150 mg/L Au(III) solutions from $[HMIm]^+[BF_4]^-$ (measured after dilution 1000) at aqua regia after diluted 10 times (HCl: 1.2 mol/L, and HNO_3:

1.6 mol/L) (V $_{[HMIm]+[BF4]-}$=1 mL, V $_{Au(III)}$ = 10 mL, T = 35℃, t = 6 h).

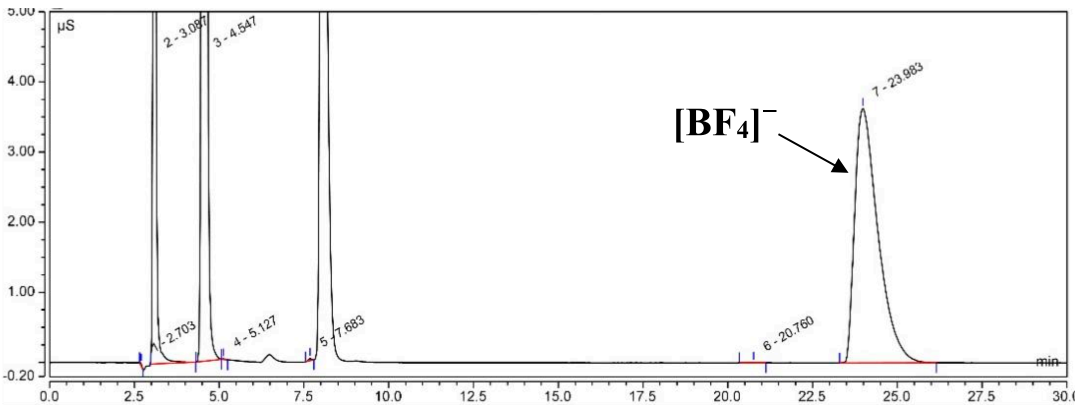

(**k**) The supernatant after extraction of the 300 mg/L Au(III) solutions from $[HMIm]^+[BF_4]^-$ (measured after dilution 1000) at aqua regia after diluted 10 times (HCl: 1.2 mol/L, and HNO_3: 1.6 mol/L) (V $_{[HMIm]+[BF4]-}$=1 mL, V $_{Au(III)}$ = 10 mL, T = 35℃, t = 6 h).

3.2 The concentration of $[BF_4]^-$ in water under different conditions

Supplementary Table S1. The concentration of $[BF_4]^-$ in water under different conditions

	$c_{(Au(III))}$	Adsorption		Solvent extraction	
		S	$c_{[BF4]-}$ in aqueous phase	S (D=1000)	$c_{[BF4]-}$ in aqueous phase
pH = 2	150ppm	0.013	0.122	1.927	18040
	300ppm	0.023	0.215	1.875	17550
Dilute 10 times of aqua regia(HCl：1.2 mol/L, and HNO_3: 1.6mol/L)	150ppm	0.209	1.96	2.841	26600
	300ppm	0.201	1.89	2.855	27010

S: peak area, D: Dilution times.

4. Effect of metal salts on Au(III) adsorption

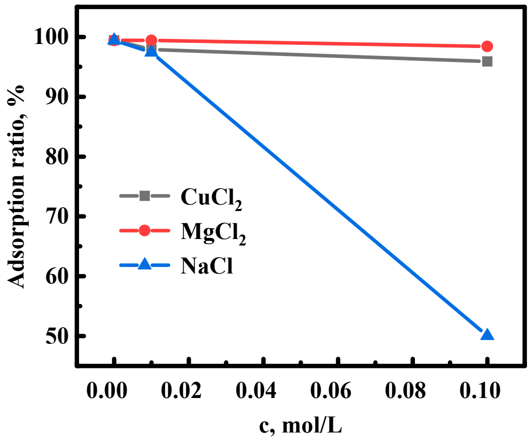

Supplementary Figure S3. Effect of metal salts on Au(III) adsorption ($c_{(Na+,Mg2+,Cu2+)}$=0, 0.01, and 0.1 mol/L, $c_{Au(III)}$=60 mg/L, $V_{Au(III)}$=20 mL, $m_{[HMIm]+[BF4]-@UiO-66}$=10 mg, t=6 h, T = 35℃, pH = 2).

5. Effect of the initial concentration of Au(III) on the adsorption amount of UiO−66

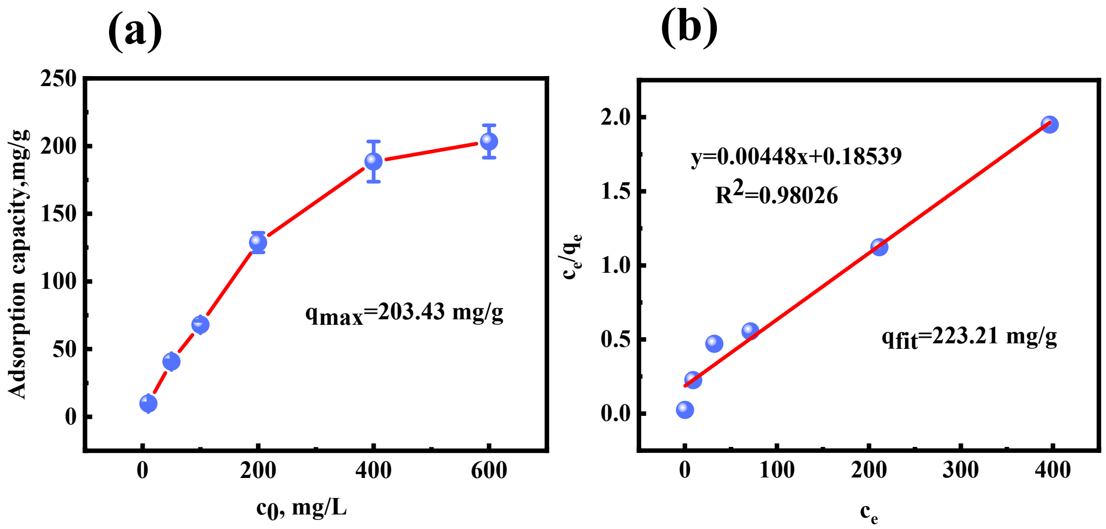

Supplementary Figure S4. (**a**) Effect of the initial Au(III) concentration on adsorption capacity of UiO−66 ($V_{Au(III)}$=10 mL, m_{UiO-66}=10 mg, t=6 h, T = 35℃, pH = 2); (**b**) Langmuir model fitting.

6. The validation studies of Atomic Absorption Spectroscopy (AAS) and Ion Chromatography (IC)

6.1 The validation studies of AAS

Supplementary Table S2. Method parameters for determination of metal ions by AAS.

Metal ions	linearity range	Characteristic concentrations (mg/L)	limits of detection (mg/L)	precision
Au	0−10 mg/L	0.059	0.016	0.44%

Mg	0–0.4mg/L	0.022	0.003	0.79%
Cu	0–5 mg/L	0.019	0.003	0.25%
Zn	0–0.5 mg/L	0.006	0.002	0.36%
Pb	0–15 mg/L	0.141	0.029	0.98%
Ni	0–3 mg/L	0.041	0.008	0.67%
Fe	0–5 mg/L	0.042	0.017	0.83%

Supplementary Table S3. Method parameters for determination of $[BF_4]^-$ by IC.

linearity range	limits of detection (mg/L)	Limits of quantification (mg/L)	precision
0–500 mg/L	0.16	0.6	0.17

The standard solutions of 0, 0.5, 1, 2, 10, 50, 100, 150, 200, and 500 mg/L $[BF_4]^-$ were configured and determined. The standard solutions were diluted a certain number of times and then sampled for analysis. The detection limits and quantitation limits were examined when the signal–to–noise ratio (S/N) was 3 and 10, respectively. Precision was calculated by measuring $[BF_4]^-$ standard solution (50 mg/L) for 8 consecutive times under optimal conditions of the instrument.

7. The technical details of AAS analytical experiments

Supplementary Table S4. The technical details for determination of metal ions by AAS.

Metal ions	absorption wavelengths (nm)	Spectral bandwidth(nm)	Burner height(nm)	Air pressure (Mpa)	C_2H_2 (Mpa, mL/min)
Au	242.8	0.4	6	0.2	0.05, 1300
Mg	285.2	0.4	6	0.24	0.05, 1500
Cu	324.7	0.4	5	0.22	0.05, 1600
Zn	213.9	0.4	6	0.24	0.05, 1300
Pb	283.3	0.4	6	0.24	0.05, 1500
Ni	232.0	0.2	6	0.24	0.05, 1300
Fe	248.3	0.2	10	0.22	0.05, 2300
